# Late weaning is associated with increased microbial diversity and *Faecalibacterium prausnitzii* abundance in the fecal microbiota of piglets

**DOI:** 10.1186/s42523-020-0020-4

**Published:** 2020-01-16

**Authors:** Francesca Romana Massacci, Mustapha Berri, Gaetan Lemonnier, Elodie Guettier, Fany Blanc, Deborah Jardet, Marie Noelle Rossignol, Marie-José Mercat, Joël Doré, Patricia Lepage, Claire Rogel-Gaillard, Jordi Estellé

**Affiliations:** 1 GABI, INRA, AgroParisTech, Université Paris-Saclay, 78350 Jouy-en-Josas, France; 2 Department of Agricultural and Food Sciences, University of Bologna, Bologna, Italy; 3 Istituto Zooprofilattico Sperimentale dell’Umbria e delle Marche ‘Togo Rosati’, Perugia, Italy; 4 ISP, INRA, Université Tours, Nouzilly, France; 5 UE PAO, INRA, Nouzilly, France; 6 IFIP-Institut du porc and Alliance R&D, Le Rheu, France; 7 MICALIS, INRA, AgroParisTech, Université Paris-Saclay, Jouy-en-Josas, France; 8 MetaGenoPolis, INRA, Université Paris-Saclay, Jouy-en-Josas, France

**Keywords:** Piglet, Gut microbiota, Age, Weaning, Diversity, *F. Prausnitzii*

## Abstract

**Background:**

In pig production systems, weaning is a crucial period characterized by nutritional, environmental, and social stresses. Piglets transition from a milk-based diet to a solid, more complex plant-based diet, and their gut physiology must adapt accordingly. It is well established that piglets weaned later display improved health, better wean-to-finish growth performance, and lower mortality rates. The aim of this study was to evaluate the impact of weaning age on fecal microbiota diversity and composition in piglets. Forty-eight Large White piglets were divided into 4 groups of 12 animals that were weaned at different ages: 14 days (early weaning), 21 days (a common weaning age in intensive pig farming), 28 days (*idem*), and 42 days (late weaning). Microbiota composition was assessed in each group by sequencing the 16S rRNA gene using fecal samples taken on the day of weaning, 7 days later, and at 60 days of age.

**Results:**

In each group, there were significant differences in fecal microbiota composition before and after weaning (*p* < 0.05), confirming that weaning can drastically change the gut microbiota. Microbiota diversity was positively correlated with weaning age: microbial alpha diversity and richness were higher in piglets weaned at 42 days of age both on the day of weaning and 7 days later. The abundance of *Faecalibacterium prausnitzii* operational taxonomic units (OTUs) was also higher in piglets weaned at 42 days of age.

**Conclusions:**

Overall, these results show that late weaning increased gut microbiota diversity and the abundance of *F. prausnitzii*, a microorganism with positive effects in humans. Piglets might thus derive a competitive advantage from later weaning because they have more time to accumulate a higher diversity of potentially beneficial microbes prior to the stressful and risky weaning period.

## Introduction

Weaning is one of the most important life transitions experienced by pigs raised for commercial meat production, and piglets go through post-weaning transient anorexia, which results in undernutrition and weight loss [1]. Indeed, it has been estimated that only 50% of piglets consume their first meal within 24 h of weaning, and 10% still have not eaten 48 h later [2]. However, piglets generally return to their pre-weaning level of energy intake 8–14 days after weaning [3]. In modern pig production systems, weaning usually occurs between the third and fourth week of life [4], and piglets are forced to switch from a highly digestible milk-based diet to a more complex, less digestible, and solid plant-based diet [1]. During this period, piglets may be afflicted with diarrhea due to gut dysbiosis and/or the colonization of the gut by enteric pathogens [1, 5]. In addition, piglets experience social stresses, such as being moved to the post-weaning building, being separated from their mothers, and being forced to live with piglets that are not their littermates [1, 6].

The swine gut microbiota comprises a large and diverse community of bacteria that play a significant role in pig health. Many recent studies have used high-throughput sequencing of the 16S rRNA gene to characterize the composition and structure of this community. In pigs, as in other mammals, the microbiota establishment begins at birth [7, 8]. From birth until weaning and then during the post-weaning period, the gut microbiota is dynamic and undergoes major compositional changes driven by age, exposure to microbes, environmental conditions, and diet [9]. Pigs bred under free-range conditions have been reported to wean between 11 and 12.5 weeks of age [10, 11] and in some cases even later (i.e., after 17 weeks [12]). Studies comparing piglet weaning ages have found that later weaning can improve health, boost wean-to-finish growth performance, and reduce mortality during the post-weaning period [13, 14]. Delaying the age at weaning in production farms has been proposed as a possible strategy for modulating and limiting the effects of weaning-associated problems [15]. However, few studies have examined how weaning age affects the early-life establishment of the pig gut microbiota and individual susceptibility to weaning-related health issues. Hence, the overall aim of this study was to characterize gut microbiota dynamics in piglets fed antibiotic-free diets and weaned at different ages.

## Results

### Effect of weaning age on piglet weight and occurrence of diarrhea

Forty-eight Large White piglets (23 females and 25 males) were divided into four groups of 12 animals that were weaned at different ages: 14 days (W14), 21 days (W21), 28 days (W28), and 42 days (W42). These groups are hereafter referred to as the weaning groups. Animals presenting diarrhea were unevenly distributed across groups, with a strong reduction in the proportion of affected animals in the groups W28 and W42: 3/10 (30%) in the W14 group, 5/12 (41%) in the W21 group, 1/12 (8%) in the W28 group, and 0/11 (0%) in the W42 group. A Chi-square test confirmed that these differences were significant (*p* < 0.05).

**Fig. 1:**
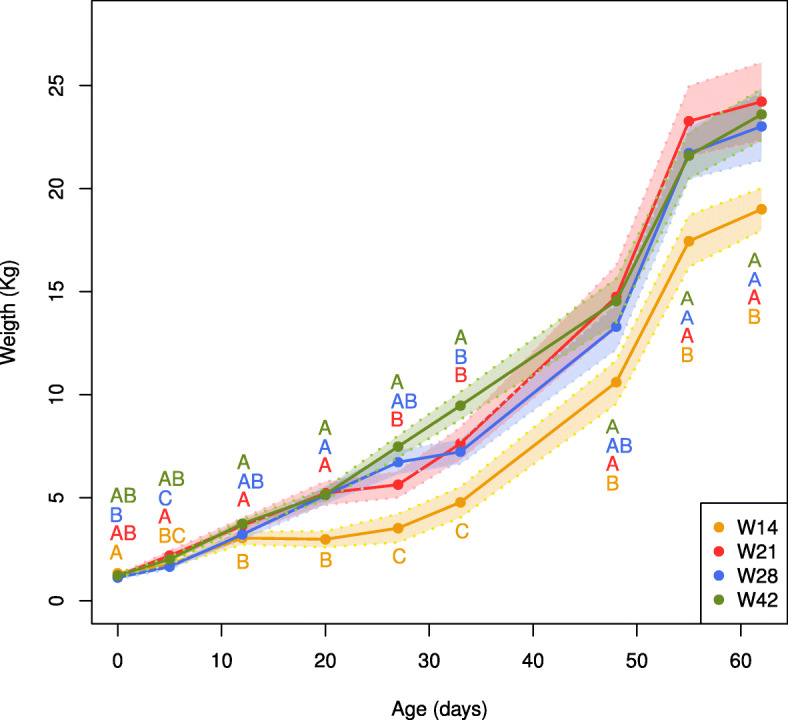
Growth curves for piglets weaned at 14 days of age (W14), 21 days of age (W21), 28 days of age (W28), and 42 days of age (W42). The solid and dashed lines show each group’s mean and standard deviation, respectively. The initial sample sizes for each group were as follows: W14: 10 animals, W21: 12 animals, W28: 12 animals, and W42: 10 animals. The samples sizes for each group after weaning were as follows: W14: 4 animals, W21: 6 animals, W28: 6 animals, and W42: 5 animals. Any statistical differences between groups are indicated by different letters in each time point, and further details can be found in Additional file 5: Table S1

To characterize piglet growth, we monitored the weight of pigs in each weaning group from birth (day 0) to 62 days of age (weight was measured at 5, 12, 20, 27, 33, 48, 55, and 62 days of age). Using ANOVAs, we found that the weaning groups differed in weight across time and that patterns of differences varied (Additional file 5: Table S1). In general, after weaning, the mean weight for the W14 group was consistently lower than the mean weights for the other groups (Fig. 1). In addition, piglets in the groups W14 (at day 20), W21 (at day 27), and W28 (at day 33) lost weight immediately after weaning. Indeed, three animals from the W14 group were euthanized because they were lethargic and failed to grow (decision made in accordance with the project’s established ethical guidelines). On day 62, the mean weights for the groups W21, W28, and W42 were statistically similar to each other, and they all differed from the weight for the W14 group (*p* < 0.05).

### Fecal microbiota sequencing, OTU identification and annotation

**Fig. 2:**
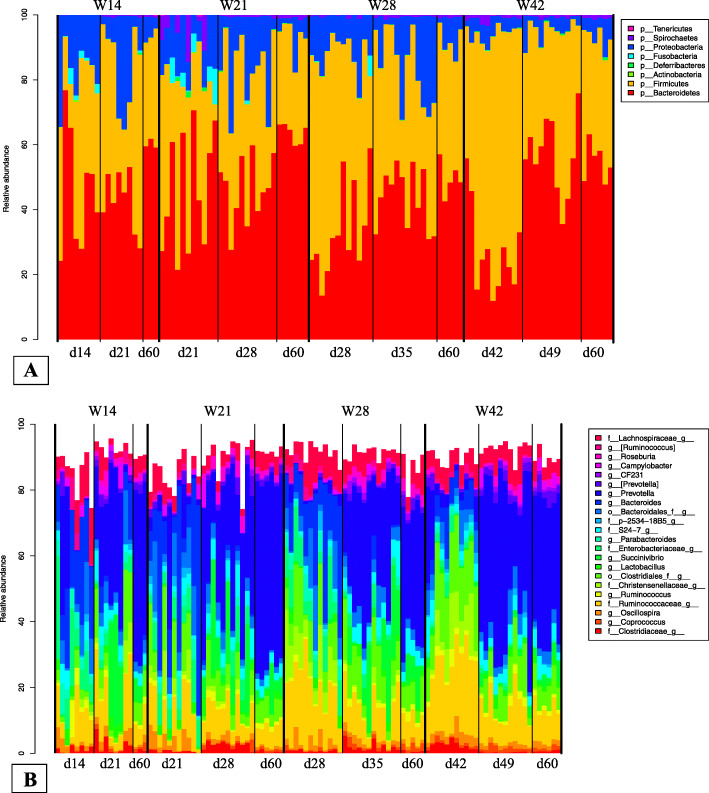
Relative abundance of the different microbial phyla (**a**) and genera (**b**) at each sampling point for every individual pig in each weaning group. Only genera present in at least 20% of the piglets are shown

The piglets’ fecal microbiota were analyzed by sequencing the bacterial 16S rRNA gene using an Illumina MiSeq Sequencer. Samples with fewer than 10,000 reads following quality control procedures were removed from the analysis, resulting in sample sizes of 3–12 piglets per sampling point (see the Methods section). After performing quality control, a mean of 63,716 reads were available for each sample. Sequences from the whole sample set were successfully clustered into 1121 operational taxonomic units (OTUs), and only 0.26% of the OTUs could not be assigned to a given phylum. Overall, 539 of the 1121 OTUs (48%) were assigned to a genus. The phyla *Firmicutes* (700/1121) and *Bacteroidetes* (340/1121) represented 62 and 30% of the OTUs, respectively. Within the phylum *Firmicutes*, 95% (665/700) of the OTUs were assigned to the order *Clostridiales*, 40% (265/665) to the family *Ruminococcaceae*, and 23% (153/665) to the family *Lachnospiraceae*. Within the phylum *Bacteroidetes*, 53% (179/340) were assigned to the genus *Prevotella.* Other phyla were also represented, but they were less common (e.g., *Proteobacteria*: 5%, *Spirochaetes*: 0.45%, *Fusobacteria*: 0.45%, *Actinobacteria*: 0.35%, *Deferribacteres*: 0.27%*,* and *Tenericutes*: 0.01%; Fig. 2a). At the phylum (Fig. 2a) and genus (Fig. 2b) levels, the overall abundance of diverse OTUs varied based on weaning age and among sampling points within weaning groups (see the following sections). When we examined the 75% most prevalent taxa in each group at the three sampling points, we found that, out of the 1121 OTUs observed overall, 760 OTUs were present in the W14 group, 807 OTUs were present in the W21 group, 882 OTUs were present in the W28 group, and 933 OTUs were present in the W42 group. This result illustrates that OTU richness increased with age at weaning.

### Effect of weaning age on fecal microbiota diversity and composition before and after weaning

**Fig. 3:**
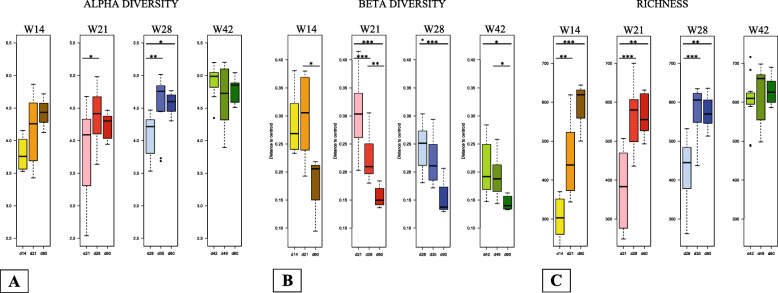
Boxplots of alpha diversity (**a**), beta diversity (**b**), and richness (**c**) for each sampling point for piglets weaned at 14 days of age (W14), 21 days of age (W21), 28 days of age (W28), and 42 days of age (W42). Any statistical differences are indicated in the figure (*p < 0.05, **p < 0.01, and ***p < 0.001)

Alpha diversity, beta diversity, and richness were calculated using the rarefied OTU counts for each group and then compared among weaning groups and sampling points (Fig. 3). ANOVAs and Tukey’s honest significant difference (HSD) tests were used to assess any resulting differences (Additional file 6: Table S2). Overall, there were significant differences (*p* < 0.05) in alpha diversity and richness among sampling points within all the weaning groups except W42. In the W42 group, only beta diversity differed significantly among sampling points. The results for alpha diversity and richness reflect the diversification that takes place in the gut microbiota during and after weaning. The results for beta diversity fit with the idea that microbiota heterogeneity declines as animals grow older. The Tukey’s HSD tests highlighted that the significant differences mainly originated from differences in diversity and richness between the pre- and post-weaning sampling points. Moreover, we observed that beta diversity declined between 7 days post weaning and 60 days of age, except in the W14 group (Fig. 3b).

**Fig. 4:**
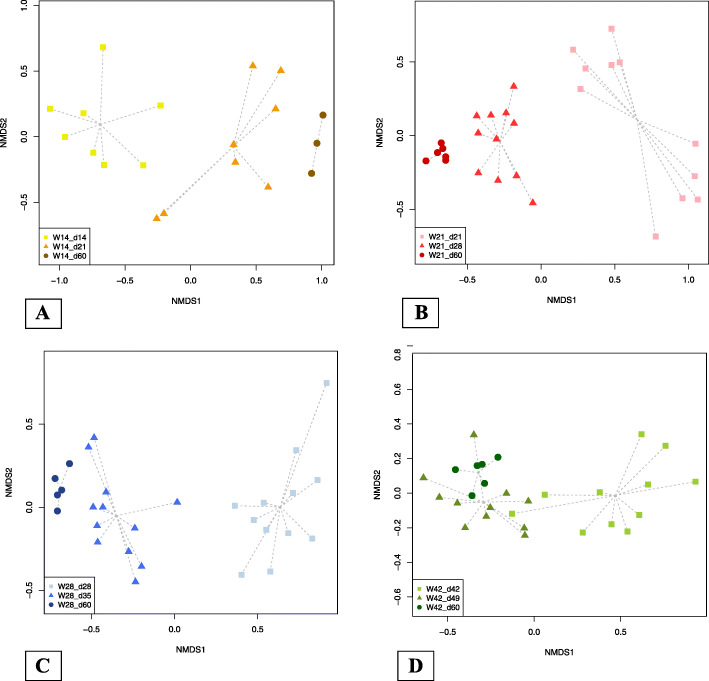
NMDS plots showing microbiota composition at each sampling point within each weaning group (**a**: piglets weaned at 14 days of age; **b**: piglets weaned at 21 days of age; **c**: piglets weaned at 28 days of age; and **d**: piglets weaned at 42 days of age)

Non-metric multidimensional scaling (NMDS) analyses were carried out using Bray-Curtis dissimilarity values quantifying overall differences in gut microbiota composition between samples collected before weaning, 7 days after weaning, and at 60 days of age for piglets in each weaning group (Fig. 4). For the groups W14, W21, and W28, there were clear differences between the results for the three sampling points. For the group W42, in contrast, the centroid for the pre-weaning data was distinct from the centroids for the data from 7 days post weaning and 60 days of age, which overlapped.

We used the metagenomeSeq package in R to identify differentially abundant (DA) OTUs within the full dataset (1121 OTUs) for each weaning group; we specifically compared the pre-weaning data and the data obtained 7 days after weaning. In the W14 group, there were 224 DA OTUs (Additional file 7: Table S3). In the W21 group, this number increased to 484 (Additional file 8: Table S4). In W28 and W42, there were 395 DA OTUs (Additional file 9: Table S5) and 461 OTUs (Additional file 10: Table S6), respectively. There was some degree of overlap among the DA OTUs (Additional file 1: Figure S1), although there were unique OTUs in all the weaning groups (W14: 44, W21: 106, W28: 71, and W42: 107). Overall, *Bacteroides*, *Ruminococcus*, *Oscillospira*, and *Clostridium* were more abundant before weaning and *Succinivibrio*, *Prevotella*, and *Campylobacter* were more abundant 7 days after weaning. Interestingly, *Faecalibacterium prausnitzii* was found to be highly abundant after weaning in all the weaning groups.

### Effect of weaning age on F. prausnitzii abundance before and after weaning

**Fig. 5:**
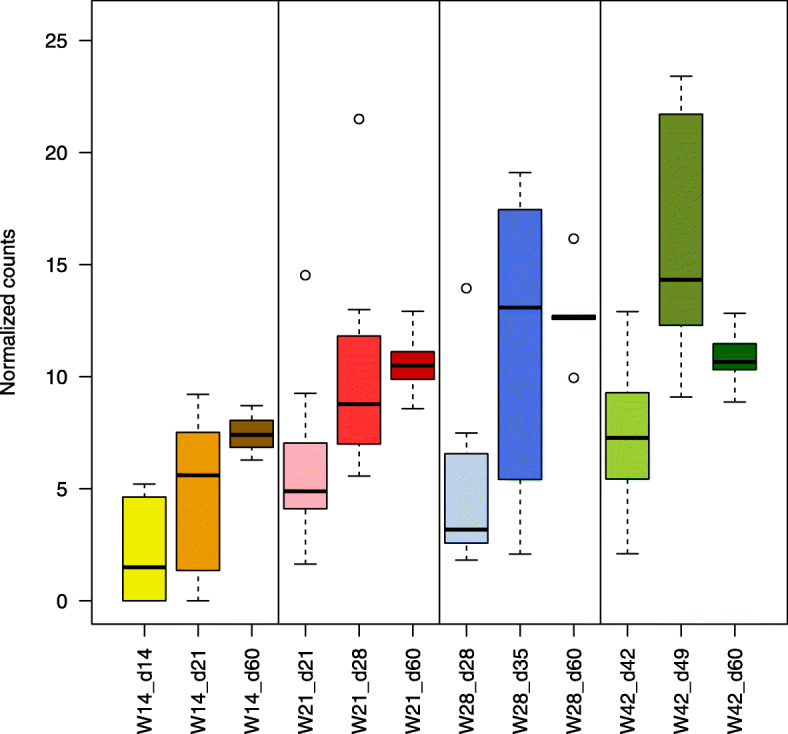
Abundance of *F. prausnitzii* at each sampling point for piglets weaned at 14 days of age (W14), 21 days of age (W21), 28 days of age (W28), and 42 days of age (W42). The normalized abundances of the three OTUs annotated as *F. prausnitzii* (OTU IDs 851,865, 350,121, and 25,215) were summed for each individual sample

In the full dataset, three OTUs were annotated as *F. prausnitzii* (OTU IDs 851,865, 350,121, and 525,215). Since at least one of these OTUs was DA in most comparisons, we decided to explore the overall abundance of *F. prausnitzii* by summing the abundances of the three OTUs for each sample. We had previously normalized these data by log scaling the cumulative sum scaling (CSS) values obtained in metagenomeSeq. For each weaning group, there was a clear increase in *F. prausnitzii* abundance over time, and the highest abundances were observed in the W42 group (Fig. 5). In the groups W14 and W21, there was a marked increase in abundance between weaning and 60 days of age; in the groups W28 and W42, abundance tended to be more stable 7 days post weaning. At weaning, *F. prausnitzii* was most abundant in the W42 group, equivalently abundant at lower levels in the W21 and W28 groups, and least abundant in the W14 group. There were significant differences among the four weaning groups (ANOVA: *p* < 0.05), and *F. prausnitzii* was more abundant before weaning in piglets weaned at a later age (Additional file 11: Table S7). Indeed, piglets weaned at 14 days of age had the lowest abundance of *F. prausnitzii* before weaning, a pattern that persisted until 60 days of age. *Post-hoc* analysis found differences in the abundance of *F. prausnitzii* between the groups W14 and W42 before weaning and between various combinations of the weaning groups at 7 days post weaning and 60 days of age (Additional file 12: Table S8).

### Effect of weaning age on fecal microbiota diversity and composition before weaning

Before weaning, alpha diversity was significantly higher in the W42 group than in the other three groups (Tukey’s HSD: *p* < 0.05) (Additional file 13: Table S9; W42 *versus* W14, W42 *versus* W21, and W42 *versus* W28). The same pattern was seen for richness, with an additional significant difference between the groups W14 and W28 (Additional file 13: Table S9). Beta diversity was only significantly different between the W42 group and the groups W14 and W21 (Additional file 13: Table S9). In the NMDS analysis, there were significant associations with litter and weaning group (*p* < 0.05) (Additional file 2: Figure S2A).

Furthermore, before weaning, there were 165 DA OTUs for the four weaning groups combined (Additional file 14: Table S10). These OTUs belonged to the phyla *Firmicutes*, *Bacteroidetes*, and *Proteobacteria* and the genera *Bacteroides*, *Ruminococcus*, and *Prevotella*. There was some overlap among groups: 44 of the DA OTUs were shared (Additional file 3: Figure S3).

Among the weaning groups, there was differential abundance of the phyla *Tenericutes*, *Spirochaetes*, *Deferribacteres*, and *Fusobacteria* (Additional file 15: Table S11) and the genera *Paludibacter, Comamonas, Helicobacter, Peptostreptococcus, Streptococcus, Treponema, Catenibacterium*, and *Dorea* (Additional file 16: Table S12).

### Effect of weaning age on fecal microbiota diversity and composition at seven days post weaning

Seven days after weaning, there was no difference in alpha diversity and richness among the four weaning groups (Additional file 13: Table S9). Beta diversity was significantly higher in the W14 group than in the other three groups, and the W42 group had the lowest beta diversity. The NMDS analysis found no differences among the groups (Additional file 2: Figure S2B). There were a total of 165 DA OTUs (Additional file 17: Table S13) that mainly belonged to the phyla *Firmicutes*, *Bacteroidetes*, and *Proteobacteria* and the genera *Prevotella*, *Ruminococcus*, *Bacteroides*, and *Oscillospira*. One of the *F. prausnitzii* OTUs was more abundant in the groups W28 and W42. The weaning groups shared 25 OTUs (Additional file 4: Figure S4), which were more heterogeneous than the OTUs shared by the groups prior to weaning; they belonged to the orders *Clostridiales* and *Bacteroidales.* In the analyses at the phylum and genus levels, only the genera *Actinobacillus, Peptostreptococcus*, and *Klebsiella* were differently abundant among the weaning groups (Additional file 18: Table S14).

### Effect of weaning age on fecal microbiota diversity and composition at 60 days of age

When the piglets were 60 days old, alpha diversity was significantly different between the groups W21 and W42 (*p* < 0.05); richness and beta diversity did not vary based on weaning age (Additional file 13: Table S9). Similarly, the NMDS analysis found no differences among weaning groups (Additional file 2: Figure S2C). There were 54 DA OTUs (Additional file 19: Table S15) that belonged to phyla *Firmicutes*, *Bacteroidetes*, and *Proteobacteria* and, for the most part, the genera *Prevotella*, *Ruminococcus*, and *Bacteroides*.

## Discussion

To the best of our knowledge, this study presents the first thorough comparison of fecal microbiota composition in piglets weaned at different ages, from 14 days (very early weaning) to 42 days (organic-like weaning). We characterized patterns of microbiota diversity and composition from just before weaning to 60 days of age and showed that piglets weaned later had time to accumulate more diverse microbial communities - which contained higher abundances of potentially beneficial bacteria like *F. prausnitzii* - before facing the difficult transition that is weaning.

Indeed, the *F. prausnitzii* OTUs were present in all the groups, regardless of weaning age, and they were significantly more abundant after weaning, when the gut microbiota diversified and matured. The abundance of the *F. prausnitzii* OTUs tracked overall alpha diversity and richness. The W14 group had the lowest abundance of *F. prausnitzii* at all the sampling points, and the W28 and W42 groups had the highest abundance after weaning. The W42 group also had the highest abundance of *F. prausnitzii* before weaning. Since we saw no signs of diarrhea in the W42 group after weaning, it migth be hypothesized that *F. prausnitzii* contributes to the resilience of weaned piglets. Indeed, based on the results for the pre-weaning period, it appeared that the later-weaned piglets (W42) had a higher abundance of *F. prausnitzii* than did earlier-weaned piglets (W14). The W14 group still had the lowest levels of *F. prausnitzii* at 60 days of age, indicating that very early weaning could have long-term effects on the abundance of this potentially beneficial species. Indeed, *F. prausnitzii* is considered to be one of the most promising next-generation probiotics (NGP) in humans because it improves gut health, notably by helping to treat inflammation-related diseases [16]. It has also been proposed that *F. prausnitzii* serves as an indicator of human intestinal health [17] because declines in its abundance have been correlated with various diseases and disorders resulting from dysbiosis [17–23]. Levels of *F. prausnitzii* are lower in patients suffering from intestinal and metabolic disorders such as inflammatory bowel disease, irritable bowel syndrome, colorectal cancer, obesity, and celiac disease, among others [16, 24–26]. *F. prausnitzii* has also been shown to have anti-inflammatory and protective effects in preclinical models of colitis [27]. Overall, these findings agree with the hypothesis that piglets could benefit from having a higher abundance of *F. prausnitzii* in their gut microbiota prior to weaning because it could provide protection against post-weaning dysbiosis and help the gut microbiota transition to a new state of gut homeostasis. To confirm this hypothesis, it will be necessary to conduct further research where sample sizes are larger at each sampling point, and also to examine a broader diversity of environmental conditions and production systems. In addition, because there are limitations associated with 16S rRNA gene sequencing and OTU assignments might not always be precise, it would be fruitful to use qPCR to quantify absolute levels of *F. prausnitzii* as well as to perform whole-metagenome sequencing to identify individual species strains.

Expanding our focus beyond *F. prausnitzii*, it has generally been shown that gut microbiota diversity and richness is positively correlated with gut health. In humans and pigs, enteric diseases, poor intestinal health, and intestinal inflammation are often associated with lower bacterial richness in the gut [28–32]. Interestingly, our results showed that piglets in the W42 group had higher alpha diversity before weaning than did piglets in the other groups, and they also had higher alpha diversity at 60 days of age than did piglets in the W14 group. Such diversity might help additionally protect gut homeostasis at weaning. Beta diversity was the lowest in the W42 group before weaning, after weaning, and at 60 days of age, meaning that piglets in this group had more homogenous gut microbiota, even early on.

Our results confirm findings from previous studies that compared the gut microbiota of piglets before and after weaning [9, 33–38]. Notably, we also observed that the phyla *Bacteroidetes* and *Firmicutes* were dominant in the fecal microbiota of weaning pigs. These two taxa accounted for more than 90% of all the sequences obtained, like in prior studies examining the ileal, cecal, and fecal microbiota of weaning and weaned pigs [9, 36, 37, 39, 40]. In piglets, the gut microbiota diversifies after weaning, and a new equilibrium of the microbiota ecosystem is established that is based on rich and stable microbial communities [7, 8]. The NMDS analysis confirmed that piglets differed in their fecal microbiota before and after weaning, which concurs with results from past research showing that weaning is associated with drastic changes in the gut microbiota that have a general impact on the intestinal ecosystem [9, 31].

We analyzed growth performance in the four weaning groups. Although there was an initial imbalance in mean birth weights among groups (animals were heavier in the W14 group), we found that weaning age affected growth: piglets in the groups W14, W21, and W28 lost weight after weaning. Post-weaning weights for the W42 group were not obtained until day 48, but its overall growth curve declined less dramatically than did the curves for the other three groups. Our results concur with those of previous studies in which weight loss was seen immediately after weaning [41, 42]. Our study showed that, even at 60 days of age, piglets in the W14 group had lower body weight than piglets in the other groups, suggesting very early weaning might have long-term effects on growth performance. In addition, the W14 group (but not the other groups) displayed morbidity after weaning, resulting in the euthanasia of three animals in accordance with the study’s ethical guidelines. Piglets in the W21, W28, and W42 groups all had more similar weights at 60 days of age, highlighting that the impact of weaning age on growth seems to be more limited after 21 days of age. Moreover, studies comparing two different weaning ages (14 days and 21 days) found that weaning age affected growth performance in a wean-to-finish facility, as well as behavioral and immunological responses to weaning and new social conditions after the nursery phase [13]. In our study, some piglets in all the groups except W42 had diarrhea, confirming that late weaning could provide protection against intestinal issues. We thus confirmed that piglets appear to be more sensitive to diarrhea when they are weaned at an earlier age [1, 5], and our results also sustain organic farming practices that promote late weaning to reduce the incidence of diarrhea [11, 12].

## Conclusions

In conclusion, our results suggest that piglet gut health could be enhanced by late weaning (i.e., at 42 days of age), as it would give the gut microbiota more time to diversity prior to weaning. Even though we looked at a relatively small number of animals from a single farm, our results fit with what has been seen in response to organic farming practices, where piglets are weaned at older ages [10–12]. Implementing late weaning in conventional production systems would be challenging since pig farms are structured to wean animals at 21 or 28 days of age. However, it may be possible to obtain the benefits of late weaning by using nutritional strategies and/or probiotics to increase microbial diversity before weaning. Indeed, our results indicate that *F. prausnitzii* could be a promising probiotic for preventing health issues related to weaning dysbiosis, and the economic loss associated to a reduced growth yield. Our results also underscore that weaning piglets are a valuable model for studying how *F. prausnitzii* might affect intestinal health in humans.

## Methods

### Study animals and phenotypes

In our study, we used 48 Large White piglets (23 females and 25 males) from 6 different litters that were bred on INRA’s experimental farm at the PAO Experimental Unit in Nouzilly (France). The piglets were randomly assigned to four groups that were weaned at different ages: 14 days (W14), 21 days (W21), 28 days (W28), and 42 days (W42). Each group included animals from two different litters to minimize block effects. At weaning, piglets were transferred into four different pens based on their litter of origin; the pens had fully slatted floors, used a flat deck system, and were temperature controlled. Six piglets from each group were euthanized 7 days after weaning to take tissue samples for a complementary study, while the others were followed until they reached 62 days of age. The quality of environmental conditions, and housing conditions were monitored throughout the study. Animals were kept in the same pen during the entire post-weaning period, and no new piglets were introduced. After weaning, piglets were fed an ad libitum standard diet of grain-based pellets, which was formulated to exceed the animals’ nutritional requirements. None of the piglets were treated with antibiotics during the experiment. Pigs were free of major pathogens and of enterotoxigenic *E. coli*, whose presence/absence was tested via PCR [43] performed on the fecal samples.

The piglets were weighed at birth and at 5, 12, 20, 27, 33, 48, 55, and 62 days of age. At the beginning of the experiment, sample sizes for each group were as follows: W14: 10 animals, W21: 12 animals; W28: 12 animals, and W42: 10 animals. After weaning, three animals in the W14 group were lethargic and failed to grow; they were therefore euthanized in accordance with the study’s ethical guidelines. Furthermore, half of the animals in each group were euthanized 7 days after weaning to collect tissues for a complementary study. On day 60, the sample sizes for each group were as follows: W14: 4 animals, W21: 6 animals, W28: 6 animals, and W42: 5 animals. During the period from weaning to 7 days after weaning, we visually scored the animals’ feces for the presence/absence of diarrhea (0 = normal feces; 1 = liquid diarrhea) (W14: 3 cases of diarrhea out of 18 observations; W21: 6/33; W28: 1/15; and W42: 0/19).

### Fecal DNA extraction and quality control

Fecal samples were collected directly from the piglets’ rectums at three different sampling points: the day of weaning, 7 days after weaning (day 21 for W14; day 28 for W28; day 35 for W28; and day 49 for W42), and at 60 days of age. Samples could only be collected from half of the animals at 60 days of age because of the earlier tissue sampling. Furthermore, in the W14 group, three piglets had been euthanized, leaving just 3 piglets to reach the age of 60 days. All the fecal samples were directly frozen in liquid nitrogen and further stored at − 80 °C until use.

A modified version of the protocol developed by Godon et al. [44] was used for DNA extraction. The method was adapted as follows to be compatible with the chemagic STAR nucleic acid workstation (Hamilton, Perkin Elmer, USA). For each sample, 200 mg of frozen fecal matter was placed in a tube and suspended in a mixture of 250 μl of guanidine thiocyanate buffer (4 M guanidine thiocyanate–0.1 M Tris [pH 7.5]), 40 μl of 10% N-lauroyl sarcosine–0.1 M phosphate buffer (pH 8.0), and 500 μl of 5% N-lauroyl sarcosine. These samples were then incubated at 70 °C for 1 h. Afterwards, a 750-μl volume of 0.1-mm-diameter silica beads (Sigma-Aldrich, Germany) was added, and the samples were shaken for 10 min at 25 agitations per second in a MM301 Mixer Mill (Retsch, Germany). The samples were subsequently centrifuged at 14,000 rpm and 4 °C for 5 min, the supernatant was collected, and 30 μl of Proteinase K (chemagic STAR DNA BTS Kit, Perkin Elmer, USA) was added. The samples were then incubated with shaking (MultiTherm Vortexer, Benchmark Scientific, USA) at 250 rpm and 70 °C; there was a final 5-min heating step at 95 °C for enzyme inactivation. Finally, the samples were again centrifuged at 14,000 rpm and 4 °C for 5 min, and the supernatant was transferred into deep-well plates for further extraction using the chemagic STAR DNA BTS Kit (Perkin Elmer, USA), in accordance with the manufacturer’s instructions (starting at the Protease K incubation step). A NanoDrop spectrophotometer (Thermo Scientific, USA) was used to assess the quality of the DNA extracts.

### Fecal DNA sequencing and bioinformatic data processing

Microbial profiling was performed via the high-throughput sequencing of the V3-V4 hypervariable region of the 16S rRNA gene (2 × 250 bp paired-end reads) using an Illumina MiSeq Sequencer (Illumina, USA). We employed the standard Illumina protocol and the primers PCR1F_343 (5′-CTTTCCCTACACGACGCTCTTCCGATCTACGGRAGGCAGCAG-3′) and PCR1R_784 (5′-GGAGTTCAGACGTGTGCTCTTCCGATCTTACCAGGGTATCTAATCCT-3′). Quality control was performed on the resulting FastQ files using FastQC software (https://www.bioinformatics.babraham.ac.uk/projects/fastqc); the files were then analyzed using QIIME software (v. 1.9.1) [45] by using the subsampled open-reference OTU picking approach [46]. Singleton OTUs and OTUs representing less than 0.005% of the total number of sequences were removed from the dataset as suggested by the software developers [47]. Chimeric sequences were identified using the BLAST algorithm and removed using QIIME. Samples with fewer than 10,000 reads after quality control procedures were eliminated from the study. On the day of weaning, 7 days after weaning, and at 60 days of age, the sample sizes were (respectively) as follows: W14: 8, 8, and 3 animals; W21: 11, 11, and 6 animals; W28: 12, 12, and 5 animals; and W42: 11, 11, and 6 animals.

### Biostatistical analyses

All our statistical analyses were performed in R (v. 3.5.1) [48]. We analyzed piglet weight using ANOVAs (*aov* function), and we assessed the frequency of piglets with diarrhea using a Chi-square test (*prop.trend.test* function). To examine microbiota diversity and composition, the biom OTU table was imported into R using the Phyloseq package (v. 1.24.2) [49]. The vegan (v. 2.5–2) package [50] was used to perform rarefaction analyses of the OTUs in each weaning group at each taxonomic level. Richness and diversity analyses were performed at the OTU level. Alpha diversity and beta diversity were calculated using the Shannon index and Whittaker’s index, respectively. Richness was defined as the total number of OTUs present in each sample. Alpha diversity, beta diversity, and log-transformed richness were then analyzed using ANOVAs (*aov* function); post-hoc comparisons were performed with Tukey’s HSD tests. We also used the vegan package to perform non-metric multidimensional scaling (NMDS): we calculated Bray-Curtis dissimilarity values and used the *metaMDS* function, which standardizes scaling, to assess differences in the overall diversity of fecal microbiota among samples. The *env_fit* function was used evaluate the statistical significance of the study variables within NMDS ordination space. These variables were sex, litter ID, piglet ID, and sampling point or weaning group. In addition, permutational multivariate analyses of variance were performed using distance matrices and the *adonis* function. The alpha level was *p* < 0.05.

OTU differential abundance testing was carried out with the metagenomeSeq package [51]. OTU counts were normalized using the cumulative sum scaling (CSS) method, and a zero-inflated Gaussian distribution mixture model (*fitZig* function) was employed to assess differences in relative OTU abundance; the significance level was set to a false discovery rate (FDR) lower than 0.05. The model accounted for the different sampling points for each weaning group, and litter effect was included as a cofactor. The overall abundance of *F. prausnitzii* was estimated by summing the log-scaled CSS normalized abundances of the three *F. prausnitzii* OTUs (OTU IDs 851,865, 350,121 and 525,215) for each sample. Differences in abundance were then evaluated using ANOVAs (*aov* function) and post-hoc comparisons were performed with Tukey’s HSD tests.

## Supplementary information

**Additional file 1: Figure S1.** Venn diagram showing the overlap in the differentially abundant OTUs before and after weaning for each weaning group.

**Additional file 2: Figure S2.** NMDS plot of microbiota composition before weaning (A), after weaning (B), and at 60 days of age (C); samples from all the weaning groups were combined.

**Additional file 3: Figure S3.** Venn diagram showing the overlap in the differentially abundant OTUs before and after weaning for each weaning group.

**Additional file 4: Figure S4.** Venn diagram showing the overlap in the differentially abundant OTUs that were more abundant after weaning in each weaning group.

**Additional file 5: Table S1.** Differences in mean weight among weaning groups and sampling points. General differences were determined using ANOVAs, and Tukey’s HSD tests were employed for post-hoc comparisons. Significant *p*-values are in bold.

**Additional file 6: Table S2.** Differences in alpha diversity, beta diversity, and richness among sampling points for each weaning group. General differences within each group were determined using ANOVAs, and Tukey’s HSD tests were employed to carry out post-hoc comparisons between sampling points. Significant *p*-values are in bold.

**Additional file 7: Table S3.** Differentially abundant OTUs before and after weaning in the W14 group.

**Additional file 8: Table S4.** Differentially abundant OTUs before and after weaning in the W21 group.

**Additional file 9: Table S5.** Differentially abundant OTUs before and after weaning in the W28 group.

**Additional file 10: Table S6.** Differentially abundant OTUs before and after weaning in the W42 group.

**Additional file 11: Table S7.** Differences in normalized *F. prausnitzii* abundances among weaning groups across all sampling points. The existence of a general difference among the groups was determined using an ANOVA, and Tukey’s HSD tests were employed to carry out post-hoc comparisons between all the groups at all the sampling points. Significant p-values are in bold.

**Additional file 12: Table S8.** Differences in normalized *F. prausnitzii* abundances among sampling points for the four weaning groups. General differences were determined using ANOVAs, and Tukey’s HSD tests were employed to compare *F. prausnitzii* abundances between weaning groups for each sampling point: before weaning, after weaning, and at 60 days of age. Significant p-values are in bold.

**Additional file 13: Table S9.** Differences in alpha diversity, beta diversity, and richness among sampling points. General differences were determined using ANOVAs, and Tukey’s HSD tests were employed for the post-hoc comparisons. Significant p-values are in bold.

**Additional file 14: Table S10.** Differentially abundant OTUs before weaning for the weaning groups.

**Additional file 15: Table S11.** Differentially abundant phyla before weaning for the weaning groups.

**Additional file 16: Table S12.** Differentially abundant genera before weaning for the weaning groups.

**Additional file 17: Table S13.** Differentially abundant OTUs after weaning for the weaning groups.

**Additional file 18: Table S14.** Differentially abundant genera after weaning for the weaning groups.

**Additional file 19: Table S15.** Differentially abundant OTUs at 60 days of age for the weaning groups.

## Data Availability

The raw sequencing data has been submitted to the NCBI’s sequence read archive (SRA) (BioProject: PRJNA540598; accession numbers SAMN11547623 to SAMN11547734).

## Abbreviations

CSS: Cumulative sum scaling
FDR: False discovery rate
HSD: Honest significant difference
NGP: Next-generation probiotics
NMDS: Non-metric multidimensional scaling
OTU: Operational taxonomic unit
PCR: Polymerase chain reaction
QIIME: Quantitative insights into microbial ecology
