# Supplementary figures and images for: Late weaning is associated with increased microbial diversity and Faecalibacterium prausnitzii abundance in the fecal microbiota of piglets
Source: Anim Microbiome. 2020 Jan 16;2:2. doi: 10.1186/s42523-020-0020-4 (PMC7807523; doi:10.1186/s42523-020-0020-4)

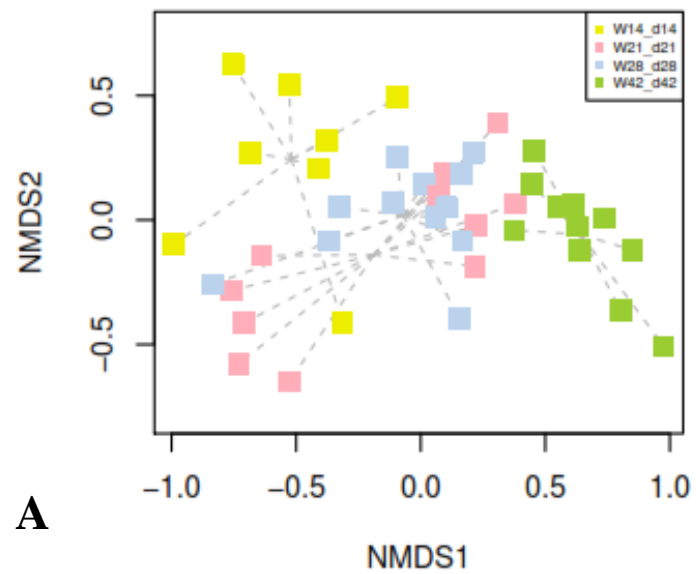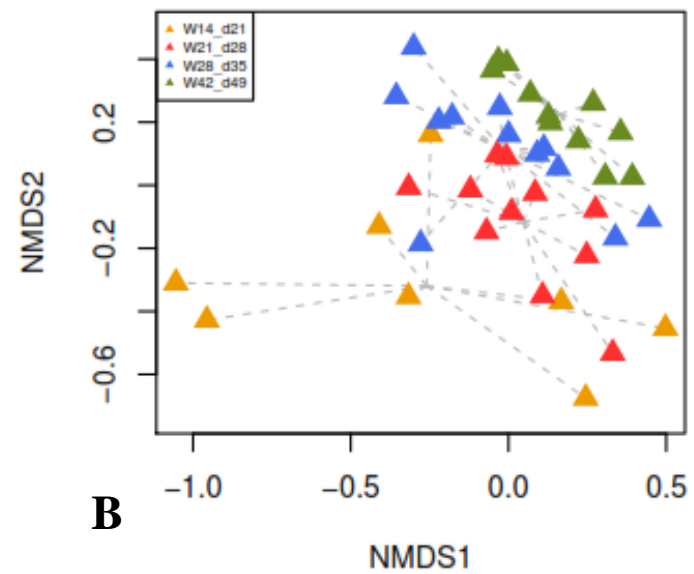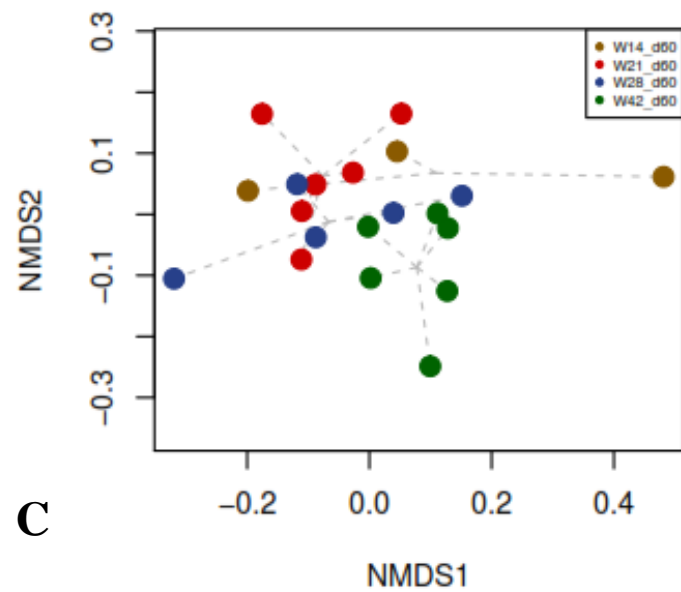

Supplement: Supplementary file 2 — Additional file 2: Figure S2. NMDS plot of microbiota composition before weaning (A), after weaning (B), and at 60 days of age (C); samples from all the weaning groups were combined. [file 42523_2020_20_MOESM2_ESM.pdf]
