# Supplementary material for: Late weaning is associated with increased microbial diversity and Faecalibacterium prausnitzii abundance in the fecal microbiota of piglets
Source: Anim Microbiome. 2020 Jan 16;2:2. doi: 10.1186/s42523-020-0020-4 (PMC7807523; doi:10.1186/s42523-020-0020-4)

Figure S3: Venn diagram showing the overlap in the differentially abundant OTUs before and after weaning for each weaning group.

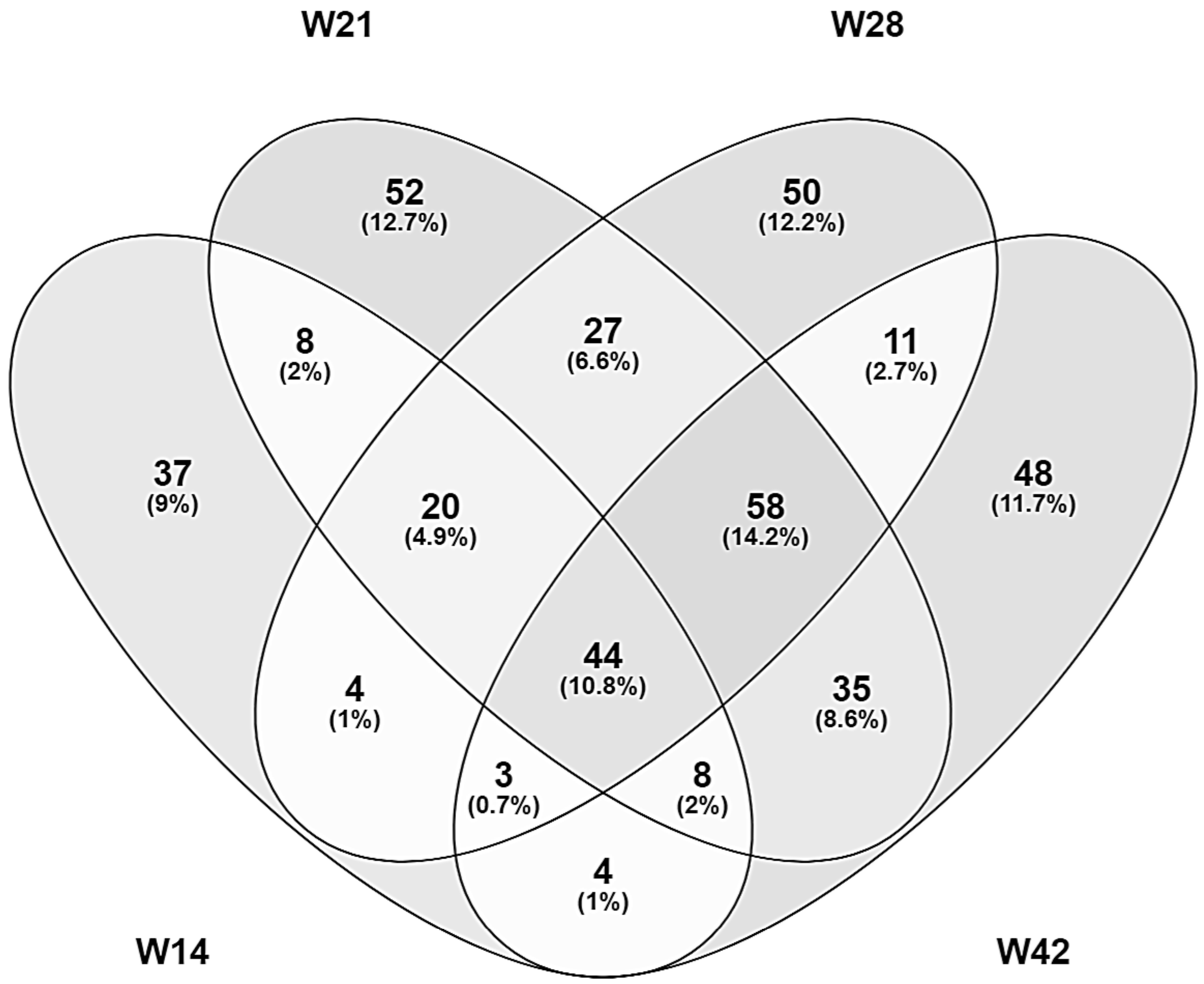

Supplement: Supplementary file 3 — Additional file 3: Figure S3. Venn diagram showing the overlap in the differentially abundant OTUs before and after weaning for each weaning group. [file 42523_2020_20_MOESM3_ESM.pdf]
