# Supplementary material for: Late weaning is associated with increased microbial diversity and Faecalibacterium prausnitzii abundance in the fecal microbiota of piglets
Source: Anim Microbiome. 2020 Jan 16;2:2. doi: 10.1186/s42523-020-0020-4 (PMC7807523; doi:10.1186/s42523-020-0020-4)
Supplement: Supplementary file 5 — Additional file 5: Table S1. Differences in mean weight among weaning groups and sampling points. General differences were determined using ANOVAs, and Tukey’s HSD tests were employed for post-hoc comparisons. Significant p-values are in bold. [file 42523_2020_20_MOESM5_ESM.docx]

|  | **Day 0** | **Day 5** | **Day 12** | **Day 20** | **Day 27** | **Day 33** | **Day 48** | **Day 55** | **Day 62** |
| --- | --- | --- | --- | --- | --- | --- | --- | --- | --- |
| **W14** | 1.333 A | 1.755 BC | 3.043 B | 2.893 B | 3.522 C | 4.777 C | 10.607 B | 17.450 B | 19.000 B |
| **W21** | 1.201 AB | 2.192 A | 3.640 A | 5.221 A | 5.362 B | 7.630 B | 14.747 A | 23.667 A | 24.212 A |
| **W28** | 1.122 B | 1.651 C | 3.213 AB | 5.125 A | 6.721 AB | 7.227 B | 13.297 AB | 21.717 A | 23.017 A |
| **W42** | 1.221 AB | 2.010 AB | 3.740 A | 5.160 A | 7.480 A | 9.467 A | 14.550 A | 21.600 A | 23.600 A |
|  |  |  |  |  |  |  |  |  |  |
| **SEM** | 0.044 | 0.077 | 0.149 | 0.228 | 0.351 | 0.428 | 0.926 | 1.040 | 1.182 |
| ***p*** | 0.0153 | <0.0001 | 0.0056 | <0.0001 | <0.0001 | <0.0001 | 0.0333 | 0.0112 | 0.0420 |

**Table S1:** Differences in mean weight among weaning groups and sampling points. General differences were determined using ANOVAs, and Tukey’s HSD tests were employed for post-hoc comparisons. Significant p-values are in bold.

SEM: standard error of the mean

A, B: Within Item, means without a common superscript differ (*p* < 0.05)
