# Supplementary material for: Late weaning is associated with increased microbial diversity and Faecalibacterium prausnitzii abundance in the fecal microbiota of piglets
Source: Anim Microbiome. 2020 Jan 16;2:2. doi: 10.1186/s42523-020-0020-4 (PMC7807523; doi:10.1186/s42523-020-0020-4)
Supplement: Supplementary file 6 — Additional file 6: Table S2. Differences in alpha diversity, beta diversity, and richness among sampling points for each weaning group. General differences within each group were determined using ANOVAs, and Tukey’s HSD tests were employed to carry out post-hoc comparisons between sampling points. Significant p-values are in bold. [file 42523_2020_20_MOESM6_ESM.docx]

**Table S2:** Differences in alpha diversity, beta diversity, and richness among sampling points for each weaning group. General differences within each group were determined using ANOVAs, and Tukey’s HSD tests were employed to carry out post-hoc comparisons between sampling points. Significant p-values are in bold.

| **Groups** | **ANOVA analysis** | | | **Tukey’s HSD tests** | | | |
| --- | --- | --- | --- | --- | --- | --- | --- |
|  | **Alpha**  **diversity** | **Beta**  **diversity** | **Richness** | **Comparison** | **Alpha**  **diversity** | **Beta**  **diversity** | **Richness** |
| W14 | 0.06 | **0.03** | **1 x 10^-4^** | d21-d14 | 0.19 | 0.88 | **1.5** **x 10^-3^** |
|  |  |  |  | d60-d14 | 0.08 | 0.06 | **2 x 10^-4^** |
|  |  |  |  | d60-d21 | 0.62 | **0.03** | 0.11 |
| W21 | **0.04** | **3 x 10^-6^** | 8 **x 10^-6^** | d28-d21 | **0.04** | **9** **x 10^-4^** | **2 x 10^-4^** |
|  |  |  |  | d60-d21 | 0.23 | 3 **x 10^-6^** | **1 x 10^-3^** |
|  |  |  |  | d60-d28 | 0.86 | **0.02** | 0.99 |
| W28 | **6** **x 10^-3^** | 4 **x 10^-3^** | 1 **x 10^-4^** | d35-d28 | **8** **x 10^-3^** | 0.2 | **2 x 10^-4^** |
|  |  |  |  | d60-d28 | **0.05** | **3 x 10^-3^** | **3 x 10^-3^** |
|  |  |  |  | d60-d35 | 0.99 | 0.07 | 0.99 |
| W42 | 0.25 | **0.01** | 0.7 | d49-d42 | 0.22 | 0.62 | 0.96 |
|  |  |  |  | d60-d42 | 0.75 | **0.01** | 0.67 |
|  |  |  |  | d60-d49 | 0.75 | **0.06** | 0.81 |
